# New insights into HIV-1-primary skin disorders

**DOI:** 10.1186/1758-2652-14-5

**Published:** 2011-01-24

**Authors:** Filiberto Cedeno-Laurent, Minerva Gómez-Flores, Nora Mendez, Jesús Ancer-Rodríguez, Joseph L Bryant, Anthony A Gaspari, Jose R Trujillo

**Affiliations:** 1 Institute of Human Virology, University of Maryland, Baltimore MD 21201, USA; 2 Department of Dermatology, Brigham and Women's Hospital, Boston MA 02115, USA; 3 Department of Dermatology, Hospital Universitario José E González. Universidad Autónoma de Nuevo León, Monterrey, NL, México; 4 Department of Pathology, Hospital Universitario José E González. Universidad Autónoma de Nuevo León, Monterrey, NL, México; 5 Department of Dermatology, University of Maryland School of Medicine, Baltimore, Maryland 21201, USA; 6 TruBios Research Institute, Johns Hopkins University, MCC, Rockville, Maryland 20850, USA

## Abstract

Since the first reports of AIDS, skin involvement has become a burdensome stigma for seropositive patients and a challenging task for dermatologist and infectious disease specialists due to the severe and recalcitrant nature of the conditions. Dermatologic manifestations in AIDS patients act as markers of disease progression, a fact that enhances the importance of understanding their pathogenesis.

Broadly, cutaneous disorders associated with HIV type-1 infection can be classified as primary and secondary. While the pathogenesis of secondary complications, such as opportunistic infections and skin tumours, is directly correlated with a decline in the CD4^+ ^T cell count, the origin of the certain manifestations primarily associated with the retroviral infection itself still remains under investigation.

The focus of this review is to highlight the immunological phenomena that occur in the skin of HIV-1-seropositive patients, which ultimately lead to skin disorders, such as seborrhoeic dermatitis, atopic dermatitis, psoriasis and eosinophilic folliculitis. Furthermore, we compile the latest data on how shifts in the cytokines milieu, impairments of the innate immune compartment, reactions to xenobiotics and autoimmunity are causative agents in HIV-1-driven skin diseases. Additionally, we provide a thorough analysis of the small animal models currently used to study HIV-1-associated skin complications, centering on transgenic rodent models, which unfortunately, have not been able to fully unveil the role of HIV-1 genes in the pathogenesis of their primarily associated dermatological manifestations.

## Review

More than 25 years have passed since the first reports on AIDS, and we are still unable to fully understand the complexity of this disease. Dermatologic disorders play a unique role in the HIV-1/AIDS spectrum, as almost all seropositive patients suffer from these debilitating and often disfiguring lesions [1]. Cutaneous disorders associated with HIV-1/AIDS undermine self-esteem and induce depression, conditions that put patients at high risk of suicide.

Since the description of Kaposi's sarcoma as an AIDS-related condition, 56 other cutaneous disorders have been linked to HIV-1/AIDS [2]. Although the introduction of highly active antiretroviral therapy (HAART) significantly decreased the prevalence of opportunistic infections and Kaposi's sarcoma, the prevalence of most inflammatory conditions primarily related to HIV-1 remains constant [3,4]. Nevertheless, while most of these dermatological manifestations directly associated with HIV-1/AIDS are currently considered as markers of disease progression, the pathogenesis of some of them is not completely understood yet [5]. Recent explanations about the pathogenesis of these disorders suggest that not only the decline in CD4^+ ^T cell counts [6], but also the shift into a Th2 cytokine profile [7], the molecular mimicry [8] and the over-expression of superantigens/xenobiotics [9], play a decisive role in the development of dermatological lesions in the context of HIV-1 infection.

Importantly, there is still a lack of conclusive evidence linking HIV-1-associated gene products to the pathogenesis of primary dermatological disorders seen in AIDS patients. This fact derives from multiple factors that include: (1) the shortage on biosafety level 3 (BSL-3) facilities and BSL-3-trained individuals; (2) the expenses associated with non-human primate studies; and (3) the lack of small animal models to study this particular disease. In order to overcome the latter, many groups, including ours, have created transgenic rodent models (rat and mouse) for the study of HIV-1-associated complications; however, none of these has successfully reproduce the data obtained from AIDS patients [10-14]. This report compiles the most recent data on the pathogenesis of inflammatory cutaneous pathology directly associated with HIV-1 infection, and discusses the reasons why transgenic animal models have failed to fully unveil the origin of many complications seen in AIDS patients.

### General immunologic cutaneous changes in the patient with HIV-1 infection

The skin is the largest and most visible organ in the body, and consequently presents the most numerous and miscellaneous types of pathological manifestations. The cutaneous immune system is unique in the fact that it contains two types of antigen-presenting cells: the Langerhans cells; and the dermal dendritic cells [15]. Both subsets of cells perform a coordinated series of events upon antigen encounter, resulting in the presentation of processed antigens to naïve T cells in the draining lymph nodes. Once activated, T cells undergo clonal expansion and home to specific sites where their expression of effector soluble factors (cytokines and chemokines) orchestrates a coordinated contention of viral, bacterial, fungal, parasitic or neoplastic invasion [16].

**Figure 1:**
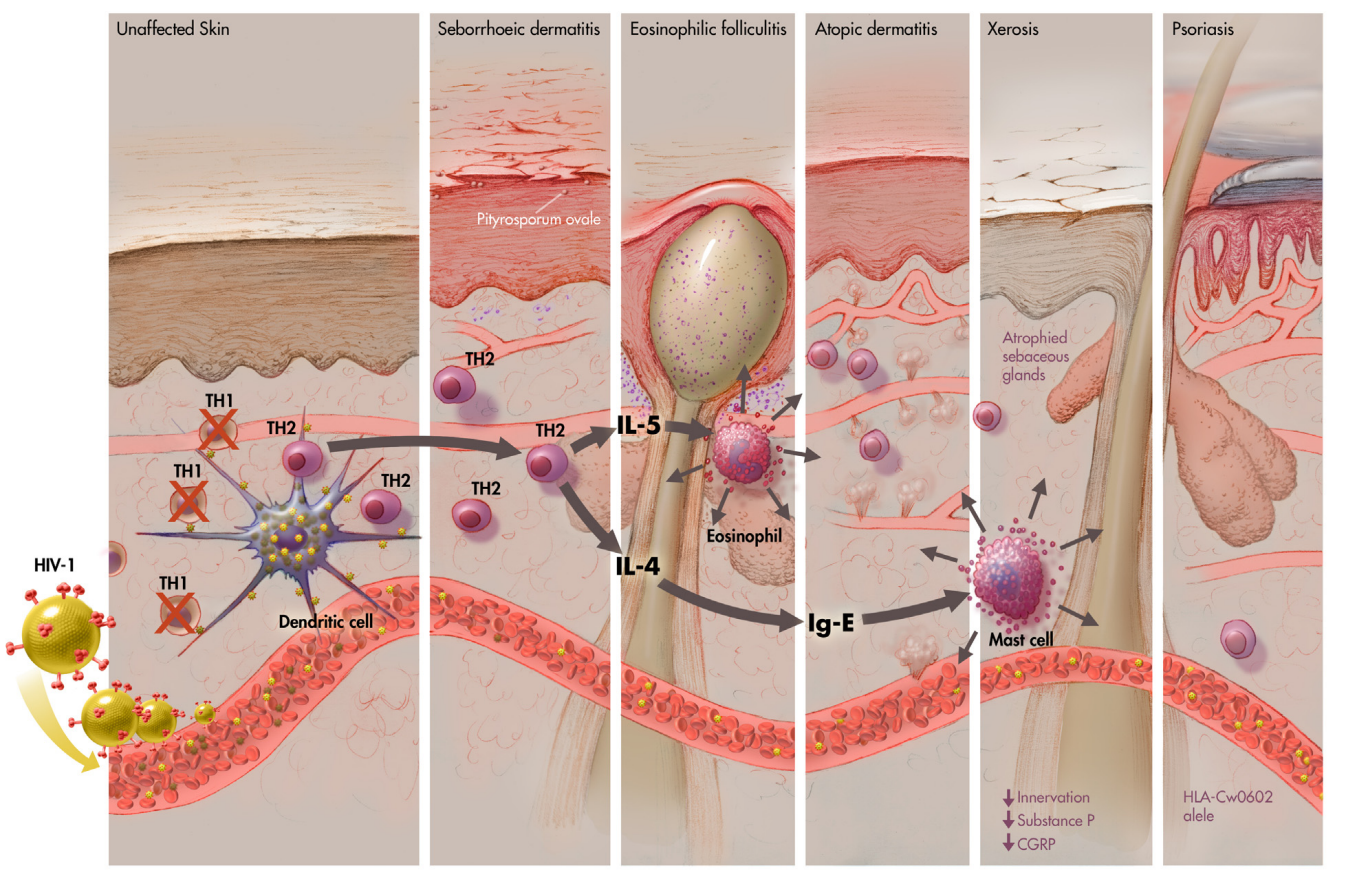
**HIV-1-driven immunological changes in the skin**. Graphic representation of the immunological processes involved in the pathogenesis of primary HIV-1 related skin disorders, highlighting the presentation of the virus by a dendritic cell to a CD4+ T lymphocyte and the subsequent changes in the cytokine profile that are brought by the death of Th1 cells.

In HIV-1-seropositive patients, the aforementioned process is abrogated in many ways. AIDS patients exhibit a marked decrease in the number and function of Langerhans cells, CD4^+^, NK cells, macrophages and monocytes [17-20] (Figure 1). While the final outcome of HIV-1 infection is the decrease in these cell types, the mechanisms by which HIV performs such lytic activities still remains controversial. Pope *et al *[21] showed that physical contact between HIV-1-pulsed dendritic cells and CD4^+ ^T cells in the context of antigen presentation promotes massive replication of the virus with a cytolytic outcome to both cells types. Moreover, the compromise of the skin-associated immune system is so critical that delayed-type hypersensitivity tests now commonly serve as monitors for the progression of the disease [22]. As a consequence of such decline in the number of antigen-presenting and CD4+ T cells, the skin becomes vulnerable to numerous opportunistic infectious agents and neoplastic disorders; however, in this article we are going to focus mainly on describing the pathogenesis of the inflammatory conditions related primarily to HIV-1 infection.

### The clinical picture

The description of skin manifestations in HIV-1/AIDS patients is complex and ranges from acne vulgaris to Kaposi's sarcoma. A brief classification of the most common dermatologic disorders in HIV-1/AIDS patients is depicted below and categorizes the spectrum of diseases in two groups: primary HIV-1-related skin disorders; and secondary mucocutaneous signs of HIV-1 infection (Table 1).

**Table 1:** Classification of HIV-1-related skin pathology

Primary Manifestations	Secondary Manifestations	
	**Infectious**	**Neoplastic**
○ Seborrheic dermatitis	○ Herpes simplex	○ Kaposi's sarcoma
○ Xerosis	○ Varicella-Zoster	○ T cell lymphoma
○ Atopic dermatitis	○ HPV infection	○ Basal cell carcinoma
○ Eosinophilic folliculitis	○ Molluscum contagiosium	○ Squamous cell carcinoma
○ Psoriasis	○ S. *Aureus *infections	
○ HIV-1-related pruritus	- Folliculitis	
○ Drug induced	- Bullous impetigo	
	- Ecthyma	
	○ Mycobacterial cutaneous infection	
	○ Bacillary angiomatosis	
	○ P. Aeruginosa cutaneous infection	
	○ Candidiasis	
	○ Dermatophyte infection	
	○ Histoplasmosis	
	○ Criptococosis	
	○ Pneumocystis	

Noteworthy, secondary manifestations of HIV-1 infection are more prevalent than primary ones. For example, in Africa, the most prevalent skin disorder in HIV-1/AIDS patients is prurigo nodularis [23-25], a pruritic condition associated with insect bites [26]. In several other countries, mucocutaneous candidiasis and herpes zoster infections are the leading cause of skin disorders in these patients [27,28].

Nevertheless, a decline of certain skin disorders in HIV-1/AIDS patients has already been observed ever since HAART became more accessible to HIV-1/AIDS patients [29]. Normal CD4^+ ^lymphocyte counts reduce the chances of suffering from co-infectious and neoplastic disorders[3]. Moreover, in developed countries, where most people have access to HAART, dermatologic conditions, such as Kaposi's sarcoma, prurigo nodularis, molluscum, and photodermatitis, are becoming rare [3,4].

However, the prevalence of most cutaneous disorders associated primarily with HIV-1 infection, as well as adverse drug reactions, have not changed in the presence of HAART [4]. This fact enhances the importance of studying the pathogenesis of these disorders in order to develop superior forms for their treatment.

### HIV-1-associated primary dermatologic disorders

#### Seborrheic dermatitis

**Figure 2:**
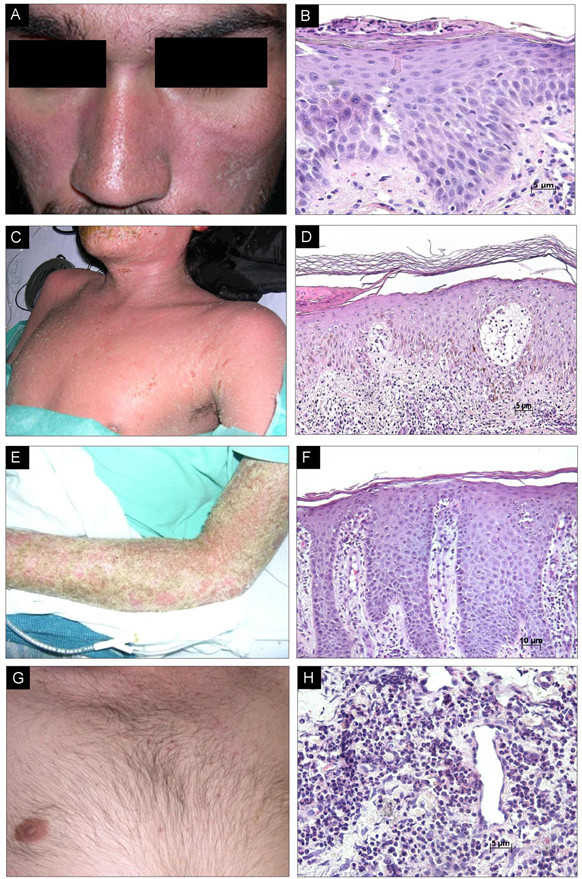
**HIV-1 primary skin disorders**. A) Patient with seborrheic dermatitis showing a papulosquamous disorder patterned on the sebum-rich areas of the scalp and face. B) Representative section (H&E 20x) shows focal parakeratosis, moderarte acanthosis, spongiosis related to hair follicles and scarce neutrophils. C) Patient with atopic dermatitis with lesions ranging from weeping crusted areas to lichenified plaques. D) Representative section (H&E 20x) shows acanthosis, mild spongiosis, and dermal infiltrate composed of lymphocytes, monocytes and few eosinophils. E) Patient with psoriasis, characterized by symmetric raised inflamed lesions covered with a silvery white scale in both lower limbs. F) Representative section (H&E 10x) shows hyperkeratosis, parakeratosis, acanthosis, spongiosis, absence of granulosum layer and neutrophil infiltrates (Munro's microabscess). G) Patient with eosinophilic folliculitis featured by follicular pustular papules on the upper part of the chest. H) Representative section (H&E 20x) shows perifollicular and perivascular infiltrate with eosinophils.

This is an entity characterized by erythema and scaling of the central part of the face, involving nasolabial folds and eyebrows, as well as the scalp [30] (Figure 2A). It is found in up to 40% of seropositive patients [30] and in up to 80% of patients with AIDS compared with 3% of the seronegative population [31,32]. Berger and Greene in 1991 [33] reported that this condition could be a hypersensitivity reaction to *Pityrosporum *yeasts, but its association is still doubtful (Figure 1). However, recent studies have failed to demonstrate any fungal overgrowth [34] or a rise in the levels of IgG titers against the yeast in HIV-1-seropositive patients [35,36].

Histologically, the lesions of seborrheic dermatitis in patients without HIV-1 show spongiform features, and with time they become less spongiotic and develop follicular plugs of orthokeratotic and parakeratotic cells, and uneven rete ridges [37] (Figure 2B). Skin biopsy specimens taken from lesions of AIDS patients show widespread parakeratosis, keratocytic necrosis, leukoexocytosis and superficial perivascular infiltrate of plasma cells [38]. These sections also show expression of heat-shock proteins (HSP65 and HSP72), a phenomenon that does not occur in HIV-1-negative patients [39]. Seborrheic dermatitis has been linked to depression of T cell function, and in patients with HIV-1 infection, it appears at early stages and worsens as the CD4^+ ^lymphocyte count declines. Thus, it can be used as a marker for disease progression [5,40].

#### Xerosis

Dryness of the skin is one of the most common skin manifestations found in patients with HIV-1 infection. This condition is found in more than 20% of people with HIV-1 [41]. It presents mainly on extremities, and it denotes one of the main causes of pruritus in AIDS patients [42]. Its pathogenesis has been suggested to include changes in the microcirculation, the nutrient supply of the skin, and in the production of sweat and oil in the skin [43]. Xerosis has also been related to certain effects on the mast cell population of the skin and to the decreased skin innervation caused by AIDS [43,44]. Reports show that such substances as calcitonin gene-related peptide and such mediators as substance P are decreased in the skin of these patients [45] (Figure 1). Xerosis has been taken by several authors as a marker for progression as it also correlates with CD4+ T lymphocyte count decline [46].

#### Atopic dermatitis

This chronic inflammatory skin condition is seen in approximately 30% to 50% of HIV-1/AIDS patients (Figure 2.C) [43,47] compared with 2% to 20% of the seronegative population [48]. This is a pruritic disorder mediated by Th2 cytokines, whose morphological features include acanthosis and spongiosis, as well as cellular infiltrates composed of lymphocytes, monocytes and eosinophils (Figure 2.D). The pathogenesis of this disorder has been linked to hypereosinophilia and to high levels of IgE secondary to Th1-Th2 imbalance [49]. Changes in cutaneous innervation and peptidergic neurotransmitters have also been related to this disease [50].

A study performed of 74 patients with atopic dermatitis showed that 53% were colonized by toxic-shock-syndrome toxin I produced by S. aureus [9]. These superantigens penetrate the skin and bind to Langerhans cells, thereby stimulating the release of IL-4 and IL-5, which further enhance the production of the allergen-specific IgE response [51]. Broadly, atopic dermatitis is thought to be initiated in predisposed individuals by a Th2 dominant cytokine production that enhances IgE release [52] (Figure 1). This Th1/Th2 cytokine imbalance is practically seen in all AIDS patients, specially in later stages, when this situation predisposes atopic manifestations [53].

#### Psoriasis

This is a chronic inflammatory skin disorder of presumed autoimmune origin found in 2% of the general population [54]. The cause of psoriasis is still under debate, but it is generally accepted to have a genetic hereditary component, and a hyperproliferative epidermal nature driven by activated lymphocytes [55]. The prevalence of psoriasis in HIV-1-seropositive individuals is similar to that of their seronegative counterparts [56]. However, psoriatic lesions in AIDS patients tend to be more severe, acral, extensive, destructive and recalcitrant [54,57] (Figures 2E and 2F). Of note, the prevalence of psoriatic arthritis is greatly increased in the HIV-1/AIDS population compared with its immunocompetent counterpart [58].

The pathogenesis of psoriasis in the context of HIV-1 infection has been associated with many immunologic events that include a decrease in the number of Langherhan's cells, but also with a potential epidermal proliferative effect of HIV-1 itself, an altered CD8:CD4 ratio and high synthesis of nitric oxide driven by HIV-1 in macrophages [56]. This association has actually led to an obscure hypothesis involving psoriasis and psoriatic arthritis with a retroviral background [59,60].

In fact, in patients with known risk for HIV-1 exposure, new onset of psoriasis may sometimes be a marker of HIV-1 infection [61]. A full comparative analysis between HIV-1-related and HIV-1-negative psoriasis is depicted in Table 2.

**Table 2:** Comparison between HIV-1 seronegative psoriasis and HIV-1 related psoriasis

Variable	HIV-1-seronegative psoriasis	HIV-1-related psoriasis	References
Frequency	1-3%	Similar to HIV-seronegative population	[54,55]
Severity	Mild-moderate	Moderate-severe	[62]
Clinical features	Erythematous plaques usually circumscribed to elbows-knees (psoriasis vulgaris)	More extensive lesions Increased presence of acral lesions and inverse psoriasis	[4,55]
Histhopatological features	Hyperproliferation and hyperkeratosis, lymphocytic infiltrate and absence of granular layer	Same	[55]
Presence of psoriatic arthritis	5-20%	23%-50%	[54,55]
Mean age of presentation	>30 years	=30 years	[63]
Family history	Frequent	Variable	[64]
Presence of HLA-Cw*0602	25%	79%	[64]
Presence of Reiter's Syndrome	Rare	Frequent	[54]
Development of erythroderma	+/+++	++/+++	[65]
Response to conventional treatment (topical steroids, Vitamin D analogues, phototherapy)	Variable	Variable-poor	[63,66]
Response to zidovudine/HAART	+/+++	++/+++	[67,68]

Expanding on the pathogenesis of psoriasis, the causative trigger of the lymphocytic activation remains unknown; however, self-antigens may play a significant role in breaking the peripheral tolerance [69]. Recently, there is growing evidence that links certain conditions of autoimmune origin to human endogenous retroviruses (HERVs) [8,70]. HERVs are genomic sequences that use reverse transcriptase and that can move from one chromosomal site to another, belonging to a class of parasitic elements that represent as much as 40% of the mammalian genome [71].

These elements were integrated into our genome million of years ago, when exogenous retroviruses infected germ cells; once integrated, these sequences were transmitted vertically as mutations of essential genes in a mendelian fashion [70]. Retrovirologists often refer to HERVs as defective proviruses with accumulated deletions, frame shift mutations, or with stop codons in *gag*, *pol *or *env *open reading frames, that limit their infectious capacity [8]. The activation of these dormant sequences of the genome has been linked to the pathogenesis of several autoimmune diseases, including most of the chronic arthropathies and systemic lupus erythematosus [72,73]. Several HERVs are expressed in normal peripheral blood lymphocytes [74], keratinocytes [75] and many other tissues [76].

Notably, the hypothesis associating HERVs with psoriasis resulted from the detection of viral-like particles resembling murine C-type retrovirus in psoriatic plaques in 1983 [77]. The microscopic findings were further supported by the detection of p27, a retrovirus-like particle in skin and lymphocytes from psoriatic patients [78,79], and more recently, by the detection of an increased titers of IgG anti-murine leukaemia virus antibodies in serum from patients with psoriasis when compared with a healthy control [80].

**Figure 3:**
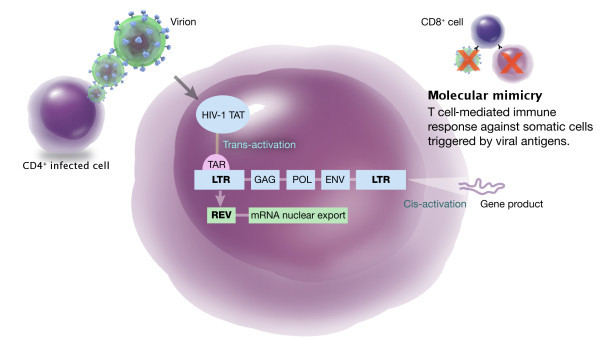
**Human endogenous retroviruses and their hypothetical role in psoriasis**. During HIV-1 infection, HIV-1 *tat *protein acts as a trans-acting factor activating HERV's long terminal repeat (LTR). Stimulation of the trans-activation region (TAR) by its interaction with the HIV-1 *tat *protein activates transcription. Subsequently, exogenous retroviruses trigger an immune response, and HERV-encoded proteins are recognized as self-antigens (molecular mimicry) awakening a cellular-based autoimmune phenomenon.

A reasonable explanation of this association involves molecular mimicry as the main phenomenon [70]. In the context of viral infection, similarities between viral amino acids and those found in host proteins lead to an autoimmune reaction mediated either by T lymphocytes or auto-antibodies that may last even after resolution of the viral infection (Figure 3) [81]. Activation of HERVs not only depend on retroviral infection, but they can also by activated in the presence of ultraviolet light or certain chemicals [69].

Recently, sequences of three different families of HERVs have been identified in psoriatic lesions [69]. Sequences of families W,E,K, and a new sequence of the ERV-9/HERV-W family were identified by the use of reverse transcriptase-polymerase chain reaction. This sequence contains at least two open reading frames that could encode for a *gag *protein and a retroviral protease. The expression of this sequence was detected in 29 of 43 lesional psoriasis skin samples, and in only two of 21 of normal skin samples [69]. Supporters of this theory consider Koebner's phenomenon as the result from the damage of keratinocytes that expose viral proteins to the immune system [70].

In addition, Mallon *et al *have suggested HIV-1-associated immune dysregulation as a possible trigger of psoriasis in those patients carrying the HLA-Cw0602 alele [62]. The HLA-Cw0602 alele might be a target for CD8 lymphocytes responding to processed peptides presented in the context of major histocompatibility complex-1, strengthening the argument for an important role for CD8 T lymphocytes in the immunopathogenesis of psoriasis.

#### Eosinophilic folliculitis

This is a cutaneous manifestation almost exclusively related to HIV-1 infection, particularly in late stages of AIDS. It was first described in 1986 [82] as a different entity from Ofuji's disease (pruritic follicular papules and pustules that involve palms and soles). Eosinophilic folliculitis (EF) presents with increased serum IgE levels, eosinophlia and peripheral leukocytosis; palms and soles are spared [83]. The most common presentation of EF is an erythematous urticarial papular rash with some pinpoint vesicles or pustules on the face, neck, and upper chest and back, almost exclusively above the nipple line [46] (Figure 2G).

Histology of the lesions shows follicular spongiosis and folliculocentric mixed inflammatory infiltrate of eosinophils, lymphocytes, hystiocytes, mast cells and neutrophils around the outer root sheaths of hair follicles [84] (Figure 2H).

EF is typically seen when CD4^+ ^cell count drops below 300 cells/mm^3 ^[85]. The suggested pathogenesis involves a Th2 cytokine response to an unknown antigen (*Pityrosporum ovale *or *Demodex folliculorum*) [86], with elevation of interleukin-4, interleukin-5 and the chemokines RANTES (chemokine that mediates chemotaxis, recruits eosinophils in the allergic late phase reaction) and Eotaxin ( a chemoattractant for eosinophils, basophils, mast cells and Th-2 lymphocytes) [7] (Figure 1). Additionally, EF has also been described as an autoimmune reaction to the sebocyte [87].

A clinical entity, called necrotizing eosinophilic folliculitis, describes the spectrum of the disease in AIDS patients who are atopic and develop ulceration, nodules and dermal follicular necrosis [88]. Its pathogenesis suggests an unrepressed Th2-type response to epicutaneous stimuli in atopic individuals [88]. EF has been interpreted as a marker of HIV-1 infection for subjects who have a high risk of developing opportunistic infections [89], but it is also part of the immune reconstitution syndrome when antiretroviral therapy is started [90].

### Miscellaneous disorders

Other dermatologic manifestations have been associated primarily with HIV-1 infection. Photodermatitis [91], vitiligo and other pigmentary alterations of the skin [92], porphyria cutanea tarda (PCT) [93], granuloma anulare [94], pityriasis rubra pilaris [95], pemphigus vulgaris and many other autoimmune reactions [96] have been reported, but a clear association between the pathogenesis of each of these disorders and the retrovirus has not yet been established.

In the case of PCT, the presence of this disorder in HIV-1/AIDS patients is thought to be secondary to a defect in the hepatic cytochrome oxidase system [97]. This impairment could lead to an aberration in porphyrin metabolism and subsequently cause porphyria [97]. Predisposing factors for the development of PCT in HIV-1/AIDS patients are co-infection with hepatitis C, alcohol abuse and hepatotoxic drug consumption [93]. Major precautions have to be taken by caregivers of these individuals as HIV-1 virions have been isolated from blister fluid of PCT/HIV-1 patients [98].

Cutaneous drug reactions (CDRs) are often reported in AIDS patients as directly related to HIV-1 infection [99]. CDRs include a wide spectrum of disorders that range from mild morbilliform reactions (~70%) to Stevens-Johnson syndrome/toxic epidermal necrolysis (7.3%) [99]. Their direct connection with HIV-1 is based on two major changes associated with the infection: the induction of defective metabolic pathways; and the modification in the immune function. HIV-1 infection induces the production of interferons [99]. Subsequently, interferons increase the production of xanthine oxidase, a superoxide that destroys the hepatic cytochrome, P-450. Modifications on this drug-metabolizing system enhance the toxic potential of many drugs [100]. Moreover, CDRs may also be stimulated by the T cell imbalance produced by HIV-1 depletion of CD4+ cells [99,101].

Stevens-Johnson syndrome (SJS), a cell-mediated immune reaction, is more prevalent in HIV-1-positive individuals than in their seronegative counterparts [99]. SJS is commonly seen as the consequence of a multi-drug regimen that includes sulfa-drugs and antiretroviral agents (e.g., nevirapine) [101,102]. Nevertheless, there are a number of case reports that support the concept of erythema multiforme as the presenting manifestation of HIV-1 seroconversion [103-105]. Yet there is not enough data to support a cause-effect relationship.

### Small animal models for the study of HIV-1-related primary cutaneous complications

Due to the high costs of non-human primate research, transgenic rodent models represent the best approach to reproduce pathologies seen in HIV-1 infection. In the late 1990s, a couple of rodent models seemed to be promising tools to study the pathogenesis of HIV-1-associated complications. These models transgenically expressed the human marker, CD4 (hCD4), and the co-receptor, hCXCR4, or the chemokine receptor, hCCR5, respectively [106,107]. As promising as they could be, numerous drawbacks were observed in these mice, which included a lack of CD4^+ ^T cells binding to HIV-1 protein gp120, and subsequent lack of infectivity and replication in the target cells [108].

From that experience, some non-infectious transgenic murine models of HIV-1 with deleted *gag *and *pol *genes were created. These HIV-1 Tg mice developed pathologic conditions similar to their human counterparts with HIV-1 infection; including the development of skin disorders [109,110]. Such lesions were reported as proliferative epidermal lesions accompanied by progressive ulceration of the epidermis, or described as benign lesions resembling papillomas, Kaposi's sarcoma-like lesions [14] or B cell lymphomas [111].

However, the data generated from these models evidenced several failures, including numerous post-entry blocks due to inefficient *tat *transactivation. The deficiency in Tat function was further correlated with its lack of interaction with a gene product encoded on human chromosome 12, named cyclin T [108]. Mice's cyclin T does not interact functionally with *tat*, a fact that makes it a non-functional viral promoter [112] and consequently an unreliable model.

In 2001, we developed an HIV-1 Tg rat that showed similar pathology to that expressed in HIV-1/AIDS patients, and that overcame some of the problems encountered in the Tg mouse [109].

Unlike mice carrying the same transgene, efficient viral gene expression occurred in lymph nodes, spleen, thymus and blood, suggesting a functional *tat *[109]. Additionally, the generation of an HIV-1 transgenic rat appears to be a much better model both from the standpoint of size and that rat-derived cells are permissive for post-entry steps in the HIV-1 replication cycle. As recently reported, the HIV-1 Tg rat developed skin lesions in about 30% of the littermates [10]. Histologically, these lesions exhibited epidermal hyperplasia and hyperkeratosis, with an intense lymphocytic infiltrate and epidermal necrosis. Additionally, while the Tg rat showed the same pattern of serum cytokines seen in HIV-1/AIDS patients with a shift from Th1 to Th2 cytokines [113], analysis of the lesional skin showed a mixed cytokine profile [10]. In fact, none of the HIV-1 Tg rodent models currently available resembles precisely the pathology observed in AIDS patients.

However, while the non-infectious HIV-1 rodent models did not reproduce similar skin pathology to that observed in AIDS patients, a recently reported humanized murine model might recapitulate the immunological phenomena seen in the skin of HIV-1-infected individuals. In this model, non-obese diabetic mice with severe combined immunodeficiency are implanted with human fethal thymic and liver organoids, followed by sub-lethal irradiation, and then transplanted with human CD34^+ ^stem cells derived from fetal livers [114]. These chimeric humanized mice show infiltration of stem cell-derived leukocytes (T and B lymphocytes) in different organs, including the liver, lung, gastrointestinal tract and grafted human skin [115]. Lately, this model has become a valuable tool to evaluate intra-rectal HIV-1 primary infection and anti-retroviral drug efficacy [114,116].

Moreover, there is another animal model that might be promising for the study of the pathogenesis of psoriasis and HERVs [117]. This mouse strain was originally reported to have a natural mutation known as flaky skin (fsn) mutation, localized in chromosome 17. This mutation induces a pathologic conditions that resembles psoriasiform dermatitis, accompanied by anaemia, hyper-IgE and anti-dsDNA autoantibodies similar to those detected in systemic lupus erythematosus [117]. The cause of this mutation is due to the insertion of an endogenous retrovirus (early transposon class) into intron 14 of the Tetratricopeptide repeat domain 7 gene. Further studies on this animal might confirm a definite role of HERVs in the pathogenesis of psoriasis.

## Conclusions

The pathogenesis of most HIV-1-related complications is not completely understood due to the complexity of this novel virus and to the drawbacks associated with their reproduction in controlled settings. Skin disorders are not the exception; the high prevalence of cutaneous manifestations related to this disease encourages us to search for more reliable explanations of the pathogenesis of these disorders.

Secondary complications related to HIV-1 are becoming less prevalent as HAART is more widely available [29]. However, no change in prevalence is seen in the primary complications. Moreover, such disorders as acne, staphylococcal infections, and erythema nodosum are being seen more frequently as part of the immune reconstitution syndrome [4].

All of the primary dermatologic complications in HIV-1-infected patients are also seen in immunocompetent patients. Such conditions as atopic dermatitis, psoriasis, and seborrheic dermatitis are extremely common dermatologic problems expressed in the general population; however, the direct role of the virus in the pathogenesis of these manifestations is still to be discovered. Noteworthy, the use of infectious and non-infectious HIV-1 Tg rodent models has failed to reproduce immunological phenomena and similar morphological skin disorders observed in AIDS patients. Better animal models, which may include humanized rodents, might represent a more suitable approach for the study of the pathogenesis of HIV-1-related disorders and the development of more effective forms of treatment.

## Consent

Written consent for publication was obtained from the patients or their relatives.

## Competing interests

All authors declare that they have no competing interests.

## Authors' contributions

FCL drafted the manuscript and conceived the outline. MGF, NM and JAR provided patient samples, photographs, helped to draft the manuscript and gave insightful comments. AAG helped to draft the manuscript and participated in its design coordination. JRT conceived the outline, helped draft the manuscript and coordinated its design. All authors read and approved the final manuscript.
